# Dietary Management of Type 2 Diabetes in the MENA Region: A Review of the Evidence

**DOI:** 10.3390/nu13041060

**Published:** 2021-03-24

**Authors:** Nahla Hwalla, Zeinab Jaafar, Sally Sawaya

**Affiliations:** Department of Nutrition and Food Sciences, Faculty of Agriculture and Food Sciences, American University of Beirut, P.O. Box 11-0236, Beirut 1107 2020, Lebanon; sallysawaya@gmail.com

**Keywords:** type 2 diabetes, dietary management, MENA region, obesity, insulin resistance, insulin glucose homeostasis

## Abstract

The alarmingly rising trend of type 2 diabetes constitutes a major global public health challenge particularly in the Middle Eastern and North African (MENA) region where the prevalence is among the highest in the world with a projection to increase by 96% by 2045. The economic boom in the MENA region over the past decades has brought exceptionally rapid shifts in eating habits characterized by divergence from the traditional Mediterranean diet towards a more westernized unhealthy dietary pattern, thought to be leading to the dramatic rises in obesity and non-communicable diseases. Research efforts have brought a greater understanding of the different pathways through which diet and obesity may affect diabetes clinical outcomes, emphasizing the crucial role of dietary interventions and weight loss in the prevention and management of diabetes. The purpose of this review is to explore the mechanistic pathways linking obesity with diabetes and to summarize the most recent evidence on the association of the intake of different macronutrients and food groups with the risk of type 2 diabetes. We also summarize the most recent evidence on the effectiveness of different macronutrient manipulations in the prevention and management of diabetes while highlighting the possible underlying mechanisms of action and latest evidence-based recommendations. We finally discuss the need to adequately integrate dietetic services in diabetes care specific to the MENA region and conclude with recommendations to improve dietetic care for diabetes in the region.

## 1. Introduction

**Figure 1:**
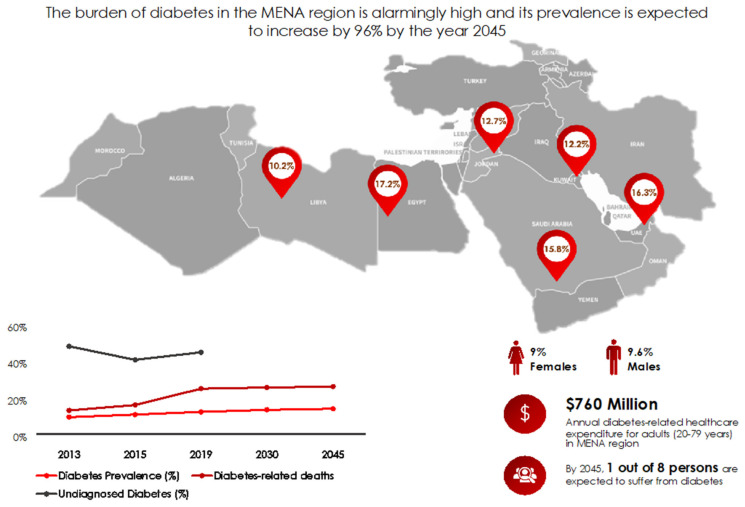
Diabetes prevalence in countries of the Middle Eastern and North African (MENA) region based on International Diabetes Federation (IDF) estimates 2019 [1]. MENA countries include Algeria, Bahrain, Egypt, Iran, Iraq, Jordan, Kuwait, Lebanon, Libya, Morocco, Oman, Qatar, Saudi Arabia, State of Palestine, Sudan, Syrian Arab Republic, Tunisia, United Arab Emirates, and Yemen.

The rising burden of diabetes mellitus is one of the major public health challenges of the current century and its prevalence among adults worldwide has more than tripled over the past 2 decades [1]. In 2010, diabetes was projected to reach around 438 million people globally by 2025. With more than five years remaining, this estimate has already been exceeded by 25 million cases with currently around 463 million people suffering from diabetes worldwide [1]. Despite the considerable scholarly and medical efforts to improve diabetes prevention, diagnosis and care, diabetes continues to impose a substantial burden on healthcare systems, societies and individuals globally, in addition to a rising death toll (11.3% of global deaths), partly due to unmanaged or poorly managed disease courses [1]. It is important to note that more than half of the adults living with diabetes are unaware of their condition which further increases their risk of developing serious health complications [1]. According to the International Diabetes Federation’s (IDF) most recent reports, the Middle Eastern and North African (MENA) region has the highest adult age-adjusted prevalence of diabetes in the world (12.2%) which is projected to increase to 13.3% by 2030, and by 2045 one out of every 8 persons is expected to suffer from diabetes [1]. Figure 1 summarizes the prevalence of diabetes in different countries of the MENA region, where Egypt, with an age adjusted prevalence of 17.2%, ranks amongst the top 10 countries with the highest diabetes prevalences worldwide. The magnitude of diabetes is especially challenging in the Gulf Cooperation Council (GCC) region where economic prosperity, driven by the energy and oil booms of the past decades, brought exceptionally rapid shifts in eating habits and dramatic rises in the levels of chronic diseases. The IDF reports that one in five people in GCC countries currently has type 2 diabetes mellitus (T2DM), which is amongst the highest prevalences worldwide. Currently, four of the top 20 countries with highest rates of diabetes per capita worldwide are located in the GCC region (United Arab Emirates (UAE), Saudi Arabia, Qatar and Bahrain) with an average prevalence of around 15% [1]. The diabetes numbers in the region are alarming by themselves but even more concerning in the midst of the current coronavirus disease 2019 (COVID-19) pandemic putting these groups at particularly higher risk to suffer and die from this emerging infection [2].

Compared to the World Health Organization (WHO) European region, the prevalence of diabetes in MENA countries is considerably higher (17% versus 12.2%) [1,2,3,4]. The rising prevalence of T2DM is influenced by a complex interchange between genetic, epigenetic and environmental factors [4]. In the MENA region, genetics may be an important contributing factor given that exclusive patterns of single nucleotide polymorphisms (SNPs) for T2DM were identified among Arab ethnicities [5]. Other considerations such as lack of health education and multiple pregnancies may also play a role in the increasing diabetes prevalence in the MENA region [6]. In addition to that, socioeconomic and demographic factors as well as older age were shown to be associated with a higher prevalence of T2DM among certain subgroups in Eastern Mediterranean countries [7]. Moreover, due to gender segregation, restrictions on outdoor activities, and compulsory wearing of full-length garments, women in the Arab world are disproportionally more sedentary than men, putting them at a particularly higher risk for obesity, insulin resistance, prediabetes, and ultimately diabetes [8,9].

A growing body of evidence associates the surge in diabetes and other non-communicable diseases with increased income per capita, economic development, technological advances, urbanization, industrialization, and expansion of global trade [10]. The most prominent factors in the MENA region are rapid urbanization, industrial development and economic growth, which have collectively and fundamentally changed the way people eat and live [3,11]. The high and increasing prevalence rates of obesity in the region with its underlying shifts in lifestyle, dietary habits and physical activity patterns have also fueled the diabetes epidemic in the region [12]. The rapid shifts in diet towards unhealthy westernized patterns, increased consumption of food, and sedentarism are amongst the characteristics of the nutrition transition phenomenon observed in the MENA region and the developing world, and are at the core of the accelerating obesity and non-communicable diseases epidemics.

The purpose of this review is to explore the mechanistic pathways linking obesity with diabetes and to summarize the most recent evidence on the association of various dietary patterns, as well as the intake of different macronutrients and food groups with the risk of T2DM. We also summarize the most recent evidence on the effectiveness of different macronutrient manipulations in the prevention and management of diabetes while highlighting the possible underlying mechanisms of action and latest evidence-based recommendations.

Electronic databases (MEDLINE, PubMed, Scopus, and Google Scholar) were searched for original research articles, meta-analyses and systemic reviews published between 2005 and 2020. International organizations and governmental websites were also explored. The main search terms were “type 2 diabetes”, “nutrition”, “diet”, “MNT”, “MENA region”, “obesity”, “nutrition transition”, “mechanism of action”, “prevention” and “management” in combination with specific terms on nutrients or dietary patterns. Publications in the last 5 years were largely selected; however, frequently referenced and highly regarded older publications were not excluded. Additionally, the selected articles’ reference lists were scrutinized, and research articles were further selected based on their relevance.

## 2. Risk Factors and Diabetes

### 2.1. Nutrition Transition and Diabetes in the Middle Eastern and North African (MENA) Region

Over the past decades, countries of the MENA region have witnessed fast rates of epidemiological and demographic changes, driven by economic development, improvements in sanitation, infrastructure and urbanization, which have significantly impacted the nutritional and health status of the populations in the region [13]. The improved standards of living have been accompanied by shifts in disease type and prevalence, that are closely related to the ongoing nutrition transition characterized by divergence from traditional healthy diets and lifestyle towards energy-rich westernized dietary patterns and sedentarism [13,14]. The Mediterranean diet is the traditional diet in many countries of the region and is widely recognized as one of the healthiest dietary patterns available. The Mediterranean diet majorly consists of fruits, vegetables, grains, extra virgin olive oil, fish, red wine, and other minimally processed foods, which are all rich sources of unsaturated fats, complex carbohydrates and fibers. The Mediterranean diet’s protective health effects have been consistently highlighted in T2DM as well as many other conditions [15,16,17]. In obese individuals, the Mediterranean diet has been shown to be correlated with greater improvement in insulin resistance compared to other nutritional interventions. Moreover, recent in vivo studies have reported encouraging results on the insulin sensitizing properties of certain nutraceuticals derived from Mediterranean foods in different insulin resistance related diseases including T2DM [18]. A recent study by Greco et al. has also shown a decrease in body weight and body mass index (BMI) and a significant increase in insulin and leptin sensitivity in obese individuals following a moderate hypocaloric Mediterranean diet [19].

Paradoxically, adherence to this dietary pattern in its native countries has been decreasing over the past decades as it is being gradually replaced by a diet rich in highly processed animal-sourced foods and simple refined carbohydrates [20,21]. A recent study by Naja et al. (2020) noted a significant decline in adherence to the Mediterranean diet among Lebanese adolescents between 1997 and 2015, from 35.03% to 27.63% respectively, using country-specific indices. This decline in adherence to the Mediterranean diet was shown to be largely driven by the decreasing consumption of whole fruits and vegetables from around 6% and 6.5% of total energy intake respectively in 1997 to around 3.4% and 3.8% respectively in 2015, and the increasing intake in parallel of “unhealthy” foods such as salty snacks and sugar sweetened beverages contributing respectively to 7.6% and 7% of adolescents’ total energy intake [22]. Gulf countries have also reported a rapid change in dietary habits and lifestyle over the past 3 decades whereby the traditional diet consisting of dates, milk, rice, brown bread, fish, and vegetables shifted to a more westernized type of diet [20]. In a recent study by Baker et al. (2020), a systematic analysis of the global and regional consumption trends of ultra-processed foods and beverages is presented. Although ultra-processed foods and beverages sales were the highest in high income regions such as North America, Europe and Australasia, significant growth in the consumption of these foods was noted in the MENA region where income per capita is rising. Notably, the consumption of vegetable oils, particularly sunflower oil, was the highest in the MENA region in 2019 compared to other regions of the world, and has increased by more than 40% since 2006 [23]. Hydrogenated vegetable oils are major sources of trans fatty acids and their increased consumption contributes to the fattening of the diet which is considered as a central feature of the western dietary pattern, and is closely associated with the development of health and metabolic issues including insulin resistance, cardiovascular diseases (CVD) and T2DM [24]. Moreover, a sub-optimal diet (e.g., low intakes of fruits, vegetables, whole grains, seafood and high intakes of processed meat, trans fats, sugar sweetened beverages) was found to be associated with cardiometabolic disease mortality, including T2DM, in 20 Middle Eastern countries [25]. There is growing awareness on the social, environmental and economic dimensions that fall behind the erosion of the Mediterranean diet in some countries of the MENA region and these include the abandonment of the traditional and cultural habits together with the globalization of food systems, and possibly the prohibitively increasing cost of certain components of this diet which was once considered to be the diet of the poorest societies [26,27]. The scale of the dietary change in the region highlights the importance of monitoring these changes, developing population wide interventions and strategies to preserve the traditional dietary patterns of the region, and mitigating the looming health threats including that of the diabetes epidemic.

### 2.2. Obesity and Diabetes

Obesity is a primary etiological contributor in the development of diabetes and is by far the leading risk factor behind the rising prevalence of diabetes worldwide [6]. Over the past few decades, the MENA region has witnessed major economic, social, lifestyle, and political changes that have potentially contributed to the rise in obesity prevalence [28]. The significant association between diabetes and obesity has been documented in several countries of the MENA region [29,30,31]. In Saudi Arabia, Qatar, and Lebanon, around 46%, 76.3% and 36% of individuals with diabetes were respectively found to be also obese [32,33,34]. In Kuwait, the prevalence of T2DM among obese males and females was noted to be around 48% and 77%, respectively [35]. In Bahrain, after adjusting for all factors, obesity was the found to be the strongest risk factor for developing diabetes [36]. Indeed, obesity is thought to account for 80–85% of the risk of developing T2DM [37]. Moreover, compared to non-obese counterparts, obese diabetic patients are at higher risks for microvascular complications, worse diabetes prognosis, and death [38].

**Figure 2:**
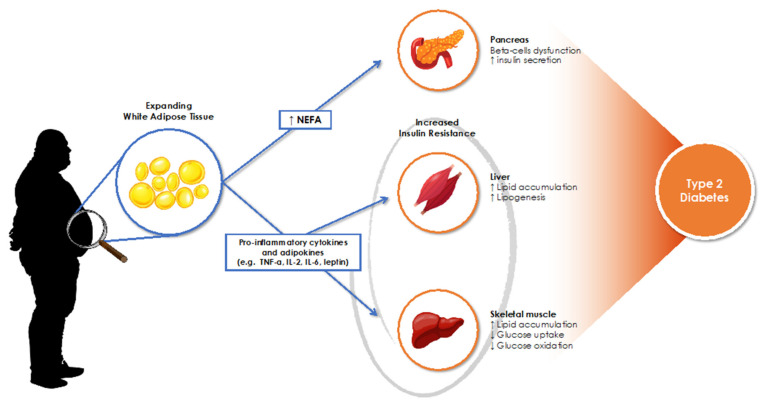
Overview of mechanistic links between obesity and type 2 diabetes. NEFA: Non-esterified fatty acids. Large arrows indicate influence.

The understanding of the mechanistic links between obesity and diabetes has grown over the past decades and a number of factors relating these two conditions have been identified including insulin resistance, pro-inflammatory cytokines, endothelial dysfunction, dysregulated lipid metabolism, and mitochondrial dysfunction among others [39,40,41]. The interaction between these pathways is highly complex and their relative significance is far from being completely uncovered. The influence of obesity on the risk of CVD and diabetes is not only determined by the degree of obesity, but more potently by individuals’ body composition and fat distribution [41,42]. Visceral adiposity in particular, reflected in a high waist to hip ratio, has been closely linked to increased risk of metabolic disorders including hyperinsulinemia and T2DM [43]. Effectively, the association between abdominal obesity and diabetes has been observed in Egypt [44], Jordan [45], Iran [46], Iraq [47], Oman [48] and Saudi Arabia [49]. In obesity, the accumulation of fat and the excessive expansion of the white adipose tissue in the abdominal area induce an inflammatory response and the release of adipokines and pro-inflammatory cytokines (e.g., Tumor Necrosis Factor-α, Interleukin-6), which decreases the expression of insulin receptors at the levels of adipocytes, hepatocytes and skeletal muscles cells, leading to insulin resistance and organ dysfunction, and eventually contributing to the development of T2DM [50] (Figure 2).

The surging flow of cytokines is also known to cause a reduction in the levels of adiponectin, a hormone known to be protective against T2DM and other metabolic and cardiac conditions due to its potent anti-atherogenic effects and remarkable insulin sensitizing properties [51]. In obese and insulin-resistant models, adiponectin is consistently found to be reduced [52,53,54], although this could be explained by the state of oxidative stress in these diseases or certain genetic mutations [51,55].

The suppressed activity of insulin, known to be a major inhibitor of lipolysis, leads to increased lipolysis in adipocytes which consequently results in the production of non-esterified fatty acids (NEFA). The expanding plasma volume of NEFA further increases lipolysis, boosting NEFA release into circulation thus exacerbating the insulin-resistant state (Figure 2). High NEFA plasma levels have also been associated with decreased pancreatic beta-cells functions, essential in glucose homeostasis [56,57]. The association between elevated levels of NEFA and obesity has been firmly entrenched in the literature, however, a growing body of evidence is suggesting that this association may not be always true, as the predicted disparities between obese and non-obese individuals in NEFA levels and their effects on insulin resistance were shown to be inconsistent [50,56,58]. Whether subcutaneous fat exhibits any significant pathological effects is a yet to be proven matter that requires further investigation.

Beyond the effects of body fat distribution, the literature also distinguishes between the different subtypes of the adipose tissue as these may have different metabolic functions and play different roles in glucose homeostasis [59]. While white adipose tissue is generally involved in energy storage and associated with the inflammatory response that is responsible for the dysregulation of glucose homeostasis, the brown adipose tissue is thought to have a thermogenic role, dissipating heat and contributing to the regulation of body temperature and weight [60]. The process of differentiation of beige adipocytes within white adipose tissue, also known as browning, has become a key focus area for research against diabetes and obesity due to brown adipose tissue’s fat-burning and heat-producing potential [61]. Further research may elucidate additional pathophysiological mechanisms and common pathways connecting obesity and T2DM which could become therapeutic targets addressing issues of excess weight gain and diabetes control concurrently.

One of the most important underlying etiological pathways common to both obesity and T2DM is increased exposure to unhealthy foods exceeding energy needs. While diabetes is mostly treated using pharmacotherapy, dietary and lifestyle interventions remain a cornerstone of effective treatment strategies.

## 3. Dietary Management of Diabetes

### 3.1. Significance and Barriers

Nutrition and lifestyle interventions are acknowledged as integral components of successful T2DM management plans. Compelling evidence supports the effectiveness and cost-effectiveness of medical nutrition therapy (MNT) administered by licensed dietitians in improving clinical outcomes and quality of life of diabetic patients [62,63,64]. A rich body of evidence corroborates the effectiveness of nutrition therapy as a complement to medication in reducing HbA_1_c levels in diabetic patients by up to 2% at 3 months, and sustaining this reduction on the longer term [62,65,66,67]. MNT was also shown to improve glucose tolerance, lipid profile, blood pressure, and obesity-related outcomes, and resulted in decreasing doses of glucose-lowering medications [62]. Indeed, in the Look AHEAD (Action for Health in Diabetes) trial, intensive lifestyle interventions for weight loss in patients with diabetes significantly improved glucose control and resulted in greater odds of partial remission of T2DM, as compared to a control condition of general support and education [68]. Dietary and lifestyle interventions were also shown to be crucial to prevent the progression of pre-diabetes and obesity into T2DM [62]. Notably, adherence to the Mediterranean diet in subjects at high cardiovascular risk was associated with lower diabetes incidence when compared to a low-fat diet, wherein diabetes incidence was reduced by 52%. The lower diabetes risk was also evident in the absence of significant changes in body weight and physical activity levels [69]. Additionally, findings from the MENA region (Diabetes Intervention Accentuating Diet and Enhancing Metabolism (DIADEM-1) trial)) show that intensive lifestyle interventions resulted in significant weight loss after 1 year, and were associated with diabetes remission in over 60% of participants [70].

Given the predominantly Islamic population in the MENA region, individuals with diabetes often show an intense desire to fast during the month of Ramadan, despite being exempted from fasting by the Qu’ran. However, the effects of intermittent fasting in Ramadan on glycemic control remain controversial [71]. Although some studies showed that Ramadan fasting resulted in significant reductions in fasting plasma glucose, HbA1c and BMI [72,73], other studies in the MENA region demonstrated a deterioration in glycemic control [74,75], which was more evident in patients on insulin or oral hypoglycemic agents [75,76]. Thus, it was concluded that Ramadan fasting may be deemed safe for individuals with mild and stable medical conditions however, for high-risk diabetes patients, individualized MNT is required [77,78].

Despite the proven significance and the progress in establishing evidence-based and systematic dietary guidance, the implementation of dietary management in diabetes care continues to be challenging for many reasons including the difficulty of behavioral change, and adherence to a life-long nutritional plan that may be different from the cultural diet, in addition to the lack of awareness on the importance of diet in the management of diabetes [79,80]. These barriers could be overcome with professional guidance from certified dietitians, who are trained to provide patients with practical tools to modify their diet and lifestyle and address their specific needs and targets based on individual preferences and within cultural contexts [62,81]. The involvement of dietitians with other healthcare practitioners in providing dietary counselling and management for diabetes has been previously shown to lead to better clinical outcomes including improved levels of HbA1c fasting blood glucose, cholesterol and plasma triglycerides [82,83,84,85]. Despite this fact, the under-utilization of dietetic services in diabetes management is evident in countries of the MENA region as well as worldwide [81,86,87]. In Lebanon, only 34% of T2DM patients were referred to dietetic services by their physicians which was the primary determinant for consulting a dietitian for the management of their condition [79]. Similar findings were noted in Qatar where only 17% of outpatient diabetic programs were reported to involve dietitians [88]. In the United Arab Emirates (UAE), 46% of surveyed diabetic patients reported never consulting with a dietitian since their diagnosis [87]. The suboptimal inter-disciplinary referrals and under-utilization of dietetic services in the MENA region cast serious doubts over the quality of diabetes care and underline lost opportunities to benefit from cost-effective solutions to combat the diabetes epidemic in a region where more than half of its countries are resource constrained [79].

While acknowledging the importance of dietary interventions in the management of diabetes, there has been a greater understanding of the optimal dietary advice for diabetes and the different pathways through which food may affect health outcomes including weight, lipid metabolism, and glucose homeostasis, which all directly impact the risk of diabetes and its associated complications [89]. Many dietary guidelines and systematic reviews have assessed the evidence on the best dietary approach in the management of diabetes as summarized in Table 1.

While different dietary approaches can be used to manage diabetes with varying levels of effectiveness, the Mediterranean, Dietary Approach to Prevent Hypertension (DASH) and plant-based eating patterns are shown to most consistently improve diabetes clinical outcomes in populations from different settings as shown in Table 1. The effects of these diets are thought to be directly linked to their unique combinations of foods which encourage a higher unsaturated to saturated fatty acids ratio, a lower intake of trans fatty acids, and a higher intake of dietary fibers and nutrients with anti-inflammatory properties [96,97]. In addition to that, these diets are thought to indirectly promote diabetes prevention and control through their effects on weight loss even though findings remain controversial [97]. In the MENA region, various dietary interventions for weight loss and glycemic control have shown superior results for carbohydrate-restricted, Mediterranean and American Diabetes Association (ADA) diets. However, higher quality, longer-duration and culture-sensitive studies are still needed to clearly describe an effective dietary strategy specific to the region [28]. The current dietary guidelines endorse the use of these healthy dietary patterns and recommend individualizing nutrition therapy and tailoring it to patients’ eating patterns, preferences, and metabolic goals [98].

In the below narrative, we provide an overview of the current literature on the association between different dietary components with diabetes and explore the potential mechanisms underlying their actions. We also summarize the most recent evidence on the effectiveness of dietary manipulation and different interventions in the prevention and management of diabetes. Additionally, we shed the light on the important role of nutrition therapy in T2DM prevention and management, and recommend steps to improve diabetes management in countries of the MENA region where integration of dietetics in diabetes care has been shown to be suboptimal.

### 3.2. The Metabolic Effects of Dietary Components in Diabetes

#### 3.2.1. Carbohydrates

Diabetes has long been considered as a disease of carbohydrate metabolism given its cardinal feature of hyperglycemia. Hyperglycemia is indeed the reason behind diabetes’s symptoms and associated complications such as retinopathy, neuropathy and nephropathy, and the control of blood glucose and lipid levels are primary goals in diabetes management [99].

Before insulin was developed as a treatment for hyperglycemia, reducing carbohydrate intake was the main strategy in the management of diabetes [89]. Although there is no clear consensus on a formal definition or composition, low carbohydrate diets typically consist of 60–130 g/day of carbohydrates (or 26–45% of daily energy needs), and do not seek to induce ketosis as opposed to very low carbohydrate diets which generally limit carbohydrate intake to 20–50 g per day and promote nutritional ketosis [100]. The effectiveness of low-carbohydrate diets in the management of glycemia has been extensively studied; however, findings have been inconclusive. Some studies asserted the superiority of low carbohydrate diets in glycemic control compared to other diets (usually low-fat diets), suggesting a dose-dependent relationship whereby a greater carbohydrate restriction results in better glycemic control, while others warned against the unsustainability of this approach’s effects or indicated no advantage at all [89,101,102,103,104,105]. Moreover, depending on the choice of foods that replace carbohydrates, low carbohydrate diets may be associated with adverse metabolic outcomes and micronutrient deficiencies [106]. On the other hand, a healthy, low carbohydrate, low glycemic index (GI) diet that is high in protein has shown superior micronutrient levels when compared to a conventional diet (moderate GI, moderate protein) [107]. More frequently, the inherently higher fat content of low carbohydrate diets, specifically saturated and trans-fat, has been linked to detrimental effects on lipid markers and cardiovascular health [89]. In patients with T2DM, several studies did not find an association of low carbohydrate intake with worsened lipid profile; however, longer-term studies are still needed to confirm these conclusions [108,109]. Generally, reducing dietary carbohydrate levels may result in improved clinical outcomes in the management of diabetes. Nevertheless, addressing concerns related to adherence to this kind of regimen and appropriately classifying low carbohydrate diets are required. Furthermore, additional research is needed to investigate the true effects of carbohydrate reduction on HbA1c independently of its effect on reducing requirements for diabetes medications [110]. The ADA has newly revised its lifestyle management guidelines, stating that very low carbohydrate diets are a feasible approach for those with hyperglycemia who wish to reduce glucose-lowering medications [98]. The mechanisms by which very low carbohydrate diets exert their effects was proposed to be related to the production and use of ketone bodies as more than an alternative fuel source, but as signaling molecules that may have positive physiological influences such as reducing inflammation and improving insulin sensitivity [100].

Another approach that has been explored in the management of diabetes is increasing intake of unrefined carbohydrates which some studies have found to be at least as effective as reducing carbohydrate intake in improving clinical outcomes in people with diabetes [102,111]. Owing to its usually lower fat content, a diet rich in carbohydrates has been associated with lower serum cholesterol [111]. However, evidence from large population studies indicates that the quantity of carbohydrates as a percentage of daily caloric intake is less significant than carbohydrates’ quality in determining the risk for metabolic diseases and obesity [112,113]. Whereas the consumption of foods like refined grains and sugar-sweetened beverages increases the risk, the intake of fresh and minimally processed starches, fruits and vegetables is often associated with improved clinical outcomes and a lower diabetes risk [112,114,115]. Indeed, the current guidelines assert that the evidence from isoenergetic comparisons of different carbohydrate-focused diets is inadequate to recommend an optimal carbohydrate amount or one dietary approach over others. The ADA advocates an individualized approach that matches the quantity of carbohydrate intake with habitual consumption and caloric needs, and guarantees long-term adherence.

On the other hand, a strong case could be made for the causality of certain types of carbohydrate in the development of diabetes and obesity, where the intake of refined starches and added sugars appears to be detrimental, and the intake of whole grains, legumes, fruits and vegetables is deemed protective [98].

Several systems have been in use to characterize different types of carbohydrates and evaluate their quality based on their chemical structure and properties (chain length, viscosity, type of starch), fiber content and effects on post-prandial blood glucose, with varying significance to diabetes health outcomes [112,116]. Two empirical indices are frequently used to rank carbohydrate containing foods based on their effects on post-prandial glycemia: glycemic index (GI) and glycemic load (GL) [117]. A plethora of studies have examined the clinical utility of these two measures in predicting post-prandial blood glucose and the risk of diabetes with mixed findings [118,119,120,121]. At least three mechanistic pathways relate a high GI/GL diet to T2DM which include increased glucotoxicity, lipotoxicity and increased obesity, particularly abdominal obesity, which are all known to compromise beta-cells function [122]. The relationship between GI/GL and diabetes risk had been postulated to be related to the fiber content of foods, namely cereal fiber, though a meta-analysis of 3 large prospective cohort studies found GI to be significantly associated with increased risk of T2DM, independently of the intake of cereal fiber [123]. Moreover, a recent meta-analysis by Livesey et al. (2019) based on long-term interventions studies (4–26 years) found sufficiently strong evidence to support a cause–effect relationship between GI/GL and T2DM, and to recommend their inclusion in dietary guidelines for people with diabetes. [122].

**Figure 3:**
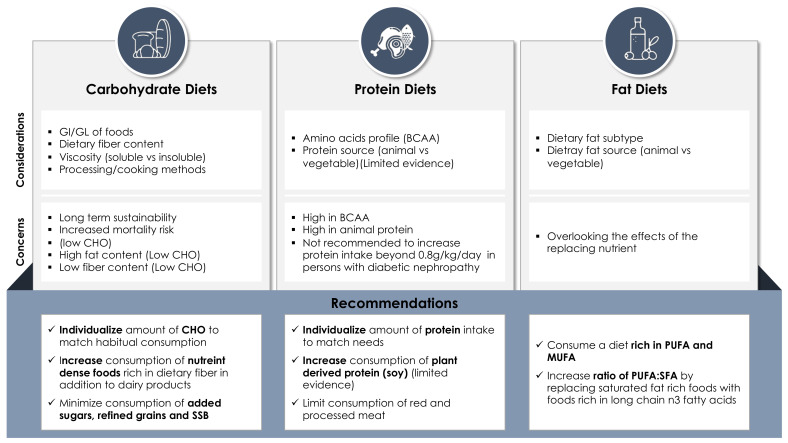
Summary of considerations, concerns and recommendations for macronutrient-focused diets in diabetes management.GI: glycemic index; GL: glycemic load; SSB: sugar sweetened beverages; PUFA: polyunsaturated fatty acids; MUFA: monounsaturated fatty acids; SFA: saturated fatty acids.

The effects of carbohydrates on post-prandial glucose are largely related to the rates of their digestion and absorption which directly impact insulin secretion, and are determined by a number of factors such as carbohydrate processing and chemical properties (e.g., viscosity, type of starch), as summarized in Figure 3.

Viscosity of a meal plays a significant role in the reduction of post-prandial glucose and is thought to induce satiety by increasing levels of glucagon like peptide 1 (GLP-1) and peptide YY (PYY), decreasing that of ghrelin, and delaying gastric emptying [116,124].

#### 3.2.2. Dietary Fiber

Post-prandial glucose may also be influenced by the presence of specific dietary ingredients. Amongst all, dietary fibers are known to have the strongest impact on the digestion and absorption of carbohydrates, and can influence post-prandial blood glucose levels primarily through their fermentation by the gut microbiota, and the production of short chain fatty acids (SCFA) [116]. The metabolic benefits of SCFAs, namely acetate, butyrate and propionate, on lipid and glucose metabolism are well-documented in the literature and include modulation of appetite via increasing hypothalamic satiety hormones, mitochondrial activity, hepatic gluconeogenesis, and lipogenesis [125]. The influence of dietary fiber on the risk of T2DM and CVD is also mediated via several other mechanisms including the modulation of body weight and reduction of inflammatory markers [116,126,127]. The effect of regularly consumed soluble dietary fiber on HbA1c in people with diabetes was reported whereby soluble fiber alone resulted in a reduction in HbA1c by about 60% [128]. In addition to the intake of soluble dietary fiber, a high intake of cereal fibers, which comprise insoluble, non-viscous and poorly fermentable fibers, has been shown to be inversely associated with the risk of T2DM in prospective studies [129,130]. In parallel, recent systematic reviews and meta-analyses on dietary fiber in diabetes management have shown that moving from lower to higher intakes of dietary fiber resulted in improved measures of glycemic control, blood lipids, body weight and inflammation, as well as a reduction in premature mortality. Nonetheless, due to the limited quantity of trials studying dietary fibers and whole grain interventions, the GRADE criteria continue to classify the evidence relating most clinical outcomes with dietary fiber as moderate, and with whole grains as low quality. Therefore, clinical trials are needed in order to strengthen the evidence base related to dietary fibers’ preventive and therapeutic effects in diabetes [131].

Despite being strongly encouraged in T2DM, the intake of fiber-rich foods (fruits, vegetables, legumes, nuts and whole grains) remains below recommendations amongst MENA populations [132]. The WHO estimated that around 85% of adults in the Arabian Gulf region do not adhere to the recommended 5 daily servings of fruits and vegetables [133]. In addition, studies from this region have highlighted the marked decrease in consumption of whole grains which are being replaced by refined cereals [134]. Indeed, low intakes of fruits and whole grains have been associated with the greatest number of diabetes and cardiometabolic deaths in 20 Middle Eastern countries, underlining the need to adopt better eating habits to halt the soaring NCD and obesity epidemics in the region [25].

#### 3.2.3. Proteins

The role of protein in T2DM often receives less attention than the other macronutrients in terms of both its metabolic and nutritional effects, although accumulating research has linked perturbations in amino acid profiling and altered protein metabolism to the risk of developing insulin resistance and diabetes [135]. The plasma levels of branched chain amino acids (BCAA) and aromatic sulfur amino acids (SAA) have been most potently implicated in metabolic diseases including diabetes, dyslipidemia and metabolic syndrome, and have been consistently found to be higher in insulin-resistant, diabetic and obese individuals compared non-diabetic or non-obese counterparts [135,136,137,138,139,140]. BCAA are primarily derived from dietary intake of foods of animal origins including meat, dairy, eggs and fish but also from plant-based products such as cereals and starches [141,142]. The observed alteration in the levels of these amino acids in obese and diabetic individuals has been postulated to result from a perturbed metabolism at the level of the adipose tissue or to be a consequence of insulin resistance which is associated with decreased expression of BCAA catabolic enzymes [143,144]. Additionally, research implicates a dietary pattern rich in animal-derived protein in contrast to a diet rich in vegetable protein which appears to exert a rather protective effect [144,145,146,147]. Similarly, dietary patterns favoring animal protein sources (beef, lamb, pork and chicken) were linked to higher mortality as compared to those favoring plant protein sources (vegetables, whole grain breads, nuts and peanut butter) [148]. In the United States, where animal-derived foods including red meat and dairy products constitute the major contributors to BCAA plasma levels, the consumption of BCAA was associated with an increased risk of T2DM as shown by several studies including a meta-analysis of 3 large prospective studies [141,146,149], although with some inconsistent findings [142,150]. Notably, this association was found to be inversely true in a Japanese cohort, where BCAA are primarily derived from cereals, fish and shellfish, highlighting a possible significant role to the quality and source of dietary protein in modulating metabolic risk and link between proteins and diabetes [151]. Moreover, the levels of total and animal-derived protein intake, but not the levels of vegetable protein, were associated with a 30% increased risk of diabetes in a 10-year follow up study [118]. Similar findings were also obtained in a more recent study showing a dietary pattern rich in plant protein to be negatively associated with the risk of diabetes as opposed to a dietary pattern where animal and red meat is the main source of dietary protein [152]. Whether the relationship between BCAA levels and metabolic risk is causative or simply correlational remains highly elusive and requires further investigation. The mechanisms underlying this association intake are also still unclear.

Beyond BCAA content, the source of dietary protein is thought to influence metabolic risk due to multiple other factors such as difference in iron and amino acid content [153,154]. Observational studies have shown an association between ferritin and T2DM suggesting a role of high iron stores in diabetes development [155]. However, this underlying association may be more complex than this simple link and has been reported to require further investigation [156]. As a pro-oxidant element, iron, namely heme iron, may catalyze certain cellular pathways that produce reactive oxygen species (ROS), thus leading to damage in cellular tissues including pancreatic B-cells. The reduction in reserves of iron has been suggested to be one of the advantages of reducing the intake of animal protein, which is the major source of the bioavailable heme iron, in contrast to plant protein which contain the less bioavailable and harder to absorb form of iron [157]. Moreover, plant proteins are thought to be higher in L-arginine, an amino acid that was found to exert beneficial effects on clinical outcomes of diabetic patients in a number of long-term RCTs [154,158,159]. Indeed, replacing around 35% of daily intake of animal protein with plant derived protein led to significant improvements in levels of HbA1C, fasting insulin and fasting glucose in diabetic patients compared to controls [154]. However, it is important to note that most of the evidence differentiating between the metabolic effects of animal and vegetable proteins are derived from prospective studies and need corroboration from randomized controlled trials [96].

The optimal quantity of protein intake is another topic of controversy. High protein diets have been advocated by diabetes experts arguing for the diet’s lower energy density and higher satiating effect favoring weight loss, and lower endogenous and exogenous insulin needs which is thought to reduce insulin induced lipogenesis and improve blood lipid markers [160]. De novo lipogenesis is typically stimulated by the intake of carbohydrates and is known to raise the levels of circulating triglycerides in the blood and promote the development of fatty liver [160,161]. Besides, a high-protein hypocaloric diet was reported to be more effective in reducing liver fat than a low protein diet, which was largely due to reductions in hepatic fat uptake and lipid biosynthesis [162]. Further arguments in support of high protein diets are related to their counteracting effects to the loss of muscle mass and sarcopenia which are associated with chronic diseases and older age [163]. However, skepticism surrounds the advantages of high-protein diets due to their higher content in animal protein and its correlation with metabolic diseases and kidney function abnormalities. Moreover, a number of studies have found that diets high in BCAA and protein in general may increase the risk of insulin resistance by blocking intra-cellular insulin signaling pathways and increasing plasma glucose levels via gluconeogenesis [136,164,165], although opposite effects were reported elsewhere [166]. On the other hand, restricting the intake of BCAA, particularly leucine, can trigger mitochondrial efficiency at the level of the adipose tissue, alter the composition of the gut microbiome in favor of SCFA producing species, and reduce insulin secretion by pancreatic beta cells without causing an increase in glucose levels, which implies improved postprandial insulin sensitivity. In a recent study by Karusheva et al. (2019), a diet low in BCAA was associated with a 20% increase in insulin sensitivity compared to a high-BCAA diet in patients with T2DM [167]. In the MENA region, the intake of BCAA and its association with risk of diabetes in a population specific context have not been evaluated before with the exception of one Jordanian study which found elevated BCAA plasma levels to be a feature of diabetic patients, congruently to other populations [168].

Despite the evidence on the role of proteins in the development and management of diabetes, current guidelines from the United States, United Kingdom and Canada do not address the risks or benefits of high protein diets [98,169,170]. The most recent ADA standards of medical care (2019) maintains the position that adjusting the amount of ingested protein beyond daily intake (which is typically 1–1.5 g/kg body weight/day or 15–20% total calories) does not influence glycemic control nor CVD risk in diabetic individuals without nephropathy. The ADA continues to recommend an individualized approach with regards to protein intake [98]. To fill the gap in guidance, the Diabetes Nutrition Study Group (DNSG) assessed the available evidence and found a protein intake of 10% to 20% of daily energy intake (%E) (or 0.8–1.3 g/kg body weight) to be safe for T2DM patients below 65 years of age, and a range of 15–20% of %E for people older than 65 years [160].

#### 3.2.4. Fats

Research published over the past decades indicates that plasma fatty acids can mediate the risk for several metabolic disorders including insulin resistance and T2DM [58]. However, the evidence for an ideal amount of total fat intake for people with diabetes is inconclusive, with fat quality appearing to be far more important than quantity. Different types of fats exert different metabolic influences on glucose-insulin homeostasis. Whereas the intake of animal derived fat, namely saturated fatty acids (SFA), and trans fatty acids (TFA), has been historically associated with detrimental cardiometabolic outcomes such as impaired insulin sensitivity, glucose intolerance, and T2DM, unsaturated fatty acids have been generally thought to be protective [93]. Existing dietary guidelines for the prevention and management of cardiometabolic diseases and diabetes generally recommend restricted intake of SFA, TFA, and cholesterol, and higher consumption of monounsaturated fatty acids (MUFA) and polyunsaturated fatty acids (PUFA) rich in omega 3. Current guidelines also recommend a diet low in total fat and animal fat, and high in vegetable fat [98,169]. Despite these guidelines being based on a plethora of observational and experimental evidence from ethnically and geographically diverse populations, the link between dietary fat consumption and cardiometabolic health continues to be one of the vexed public health issues. It appears that, although reducing dietary saturated fat was associated with a lower risk of combined cardiovascular events, it had little or no impact on diabetes diagnosis [171]. Similarly, recent meta-analyses of large long-term studies have reported an inverse association or no association of SFA with T2DM, challenging the traditional views that SFA can only lead to adverse metabolic effects [171,172,173,174,175] (Table 2).

In a meta-analysis of 16 prospective cohort studies, an inverse association was found between levels of odd-chain SFA and risk of T2DM [174]. Furthermore, higher intake of dairy products, which are rich sources of SFA, has been associated with lower diabetes risk [182,183,184].

Similar equivocal findings have been obtained on the effects of TFA in diabetes development. While the traditional longstanding view has focused on TFA’s atherogenic effects based on a considerable body of evidence associating TFA levels with negative metabolic outcomes (e.g., worse fasting glucose, fasting insulin, HbA1c, insulin resistance index) [185], more recent evidence differentiates between industrial TFA, found in processed foods, and ruminant-derived TFA, which is produced by bacterial metabolism of PUFA in ruminants’ stomach, and is correlated with decreased incidence of T2DM [173,185,186]. Due to their detrimental effects on health, the Food and Drug Administration (FDA) has recently determined that industrial TFA are no longer deemed to be generally recognized as safe (GRAS), and banned their addition to foods [96].

One reason behind the divergence in the literature on dietary fat is that foods, despite having similar fat content, include other constituents that interact within a complex food matrix that provides foods with different functionalities and behaviors than those observed when considering single nutrients in isolation [187]. Researchers have, indeed, argued that the current dietary recommendations fail to consider the food matrix and should shift focus from single nutrients to whole foods. Moreover, recommendations to reduce the intake of certain fatty acids without considering the type and composition of foods may unnecessarily restrict the intake of other nutrients that reduce the risk of T2DM and other NCDs, which may contribute to malnutrition and nutritional inadequacies, further increasing vulnerability to adverse health outcomes [188].

The effects of reducing the intake of one nutrient are also thought to be dependent on the replacing nutrient [89]. For decades, substituting SFA with PUFA has been recommended and was associated with improved metabolic outcomes. The benefits of PUFA intake, especially when in the place of SFA, are well-documented in the literature and are thought to be possibly related to PUFA’s anti-inflammatory effects, reducing inflammation, and improving insulin sensitivity. In addition to that, PUFA, particularly those of long-chains (eicosapentaenoic acid (EPA), docosahexaenoic acid (DHA), and arachidonic acid), are thought to be potent ligands and stimulators of G-protein coupled receptors (GPCR), which have direct effects on insulin secretion. The binding of PUFA to GPCR is also thought to indirectly increase the expression of GLUT-4 (glucose transporters type 4) in adipocytes and muscle cells, which increases glucose uptake [189]. In a dose-response meta-regression analysis of 102 randomized feeding trials by Imamura et al. (2016), the isocaloric replacement of SFA or carbohydrates with PUFA was associated with significant reductions of fasting blood glucose, lowered HbA1c, and improved insulin secretion capacity in patients with diabetes [176]. On a global scale, the PURE (Prospective Urban Rural Epidemiology) study, which included more than 135,000 individuals from 18 countries, found the substitution of carbohydrates with PUFA to be associated with lower mortality. Moreover, this study associated a higher intake of total fat, SFA, MUFA, and PUFA with lower total mortality, and found intake of SFA to be inversely associated with the incidence of stroke [190].

Taken together, emerging evidence raise concern over current guidelines to restrict the intake of certain fatty acids without considering the type of food and the replacing nutrient, and highlight the need to consider a whole food approach instead of a single nutrient approach in formulating dietary guidance for the management of diabetes.

#### 3.2.5. Food Groups

Recently emerging evidence have prompted a global movement towards food-based rather than nutrient-based guidelines for the prevention and dietary management of diabetes. Dietary guidelines have accordingly reformulated their recommendations to highlight the importance of limiting the consumption of specific foods and beverages that are known to be associated with an increased risk of diabetes including red and processed meats, sugar sweetened beverages (SSB), and refined carbohydrates, and increasing the intake of foods that are thought to be protective such as whole grains, fruits, vegetables, and dairy products [169,191].

The link between red and processed meats and risk of diabetes has been evaluated by multiple meta-analyses and estimated to be between 1.13 to 1.19 and 1.19 to 1.51 per 100 g per day for red meat and processed meats respectively [191,192,193,194,195,196]. Based on data from animal studies and published clinical trials, red and processed meats contain several detrimental compounds that potentially contribute to their negative effects on insulin resistance and diabetes risk, namely BCAA, advanced glycation end products (AGE), trimethylamine N-oxide (TMAO), and nitrites, which have all been closely correlated with altered glucose homeostasis and insulin resistance [195]. However, more recent meta-analyses have revealed little or no effect of red and processed meat consumption on risk of diabetes, and a very small reduction in T2DM risk with dietary patterns that are lower in red and processed meat consumption [196,197,198]. Hence, further studies are required to clarify the underlying mechanisms and investigate interactions among different dietary components in order to make firm recommendations on red and processed meat consumption [193].

The intake of SSB has been similarly strongly correlated with the risk of diabetes with a pooled effect estimate of 1.31 (95% confidence interval 1.21–1.39) [199]. The consumption of SSB acutely raises blood glucose levels, and fructose in SBB has been shown to promote hepatic de novo lipogenesis and worsen insulin resistance which increases the risk of T2DM [191,200]. In a meta-analysis of prospective studies by Schwingshackl et al. (2017), the daily consumption of risk-increasing foods (red and processed meats, SSB, and eggs) at respectively 170 g (2 servings), 105 g (4 servings), 750 mL (3 servings), 55 g (2 servings), increased diabetes risk by 3-folds compared to non-consumption of these foods. Restricting the intake of these foods was thought to decrease the risk of diabetes by around 70% [191].

Moreover, increasing the consumption of protective foods, namely whole grains, dairy products, fruits, and vegetables was inversely correlated with diabetes risk. High diet quality, as measured by Haines et al.’s Diet Quality Index [201], was associated with lower prevalence of diabetes in Iran [202]. Moreover, increasing vegetable intake was protective as compared to higher carbohydrate and meat consumption among Saudi adults [203]. In addition to that, increasing the consumption of whole grains to up to 50 g/day has been associated with a 25% decrease in diabetes risk. Several mechanisms could possibly underlie the protective effects of whole grain consumption including its association with decreased adiposity and lower fasting glucose and insulin levels [204]. Additionally, a number of nutrients such as resistant starches, soluble fibers, and phytochemicals may mediate the health effects of whole grains [205].

For the consumption of fish, a different direction in the association with diabetes risk was found based on geographical regions of the studies, whereby a strong and positive association was found for studies conducted on American populations and an inverse association was found in studies conducted on Asian populations. This regional discrepancy was highlighted in other studies as well [206,207], and could be explained by variations in the type of fish consumed, cooking methods, and levels of exposure to pollutants [206].

The association between consumption of dairy products and diabetes risk was similarly dependent on geographical region, whereby an inverse association was found in Asian and Australian but not in American or European studies [201]. In addition to that, no association was found with dairy products of high fat content in the aforementioned studies and meta-analyses [201,208]. Accordingly, it has been concluded that avoiding harmful foods and increasing the intake of protective foods could cumulatively decrease risk of diabetes by about 80% [201]. The findings of Schwingshackl et al. (2017) have been corroborated by other studies and meta-analyses [176,209,210,211].

## 4. Improving Dietetic Care for Diabetes in the MENA Region

The prevalence of diabetes in the MENA region is increasing across all countries but at remarkably staggering rates in the GCC region where economic development and income per capita is the highest compared to other Mediterranean countries (Figure 1). Relative to their wealth, GCC countries, particularly Oman, Qatar, UAE and Kuwait, appear to be substantively under-investing in NCD related research with notably greater commitment to cancer research as compared to diabetes or CVD research, despite the latter causing the most significant health burden [212]. Due to the ongoing demographic transition and economic development in the region, the at-risk of NCDs population in MENA countries is expected to grow larger, compounded by an increasing exposure to an obesogenic environment where a westernized dietary pattern is widely prevalent [212,213]. The significant contribution of this type of diet rich in sugar and processed foods to the escalating disease burden in the region has been extensively discussed in the literature and re-iterated in this review. A systematic review on the association of dietary factors and T2DM in the Middle East showed that fast food and refined grains consumption is linked to a higher risk of diabetes, whereas consumption of whole grains consumption is associated with a reduced risk [214]. Furthermore, data from Saudi Arabia revealed that consumption of an unhealthy diet significantly increased the risk of T2DM among the Saudi population. In a case-control study, the routine consumption of Kabsa (a traditional rice-based dish in Saudi Arabia), bakery items, and French fries was found to significantly increase the risk of T2DM [203]. Alternatively, a cross-sectional study in Tehran indicated that whole-grain intake was protective, whereby the highest quartile of whole-grain consumption was associated with a lower risk of T2DM [215]. In Lebanon, a case-control study evaluating the association between different dietary patterns and T2DM showed that a dietary pattern rich in refined grains, desserts, and fast-foods significantly increases the T2DM risk, while the traditional Lebanese pattern exhibits a protective effect [29]. Despite the pervasively recognized benefits of the traditional Mediterranean diet in the prevention and management of diabetes, obesity, and several other diseases, its adoption in its native countries is eroding, which constitutes an additional public health challenge for the efforts to prevent and manage NCDs epidemics. Many countries of the region have set action plans and interventions targeting dietary habits as an important modifiable risk factor in NCDs. Nevertheless, responses have been lagging and discordant with the magnitude of the problem, with many challenges that hinder effective implementation [212]. Indeed, 6 out of 15 countries in the region do not have a national strategy for diabetes, and many still do not have a national action plan against overweight and obesity which are major risk factors for NCDs [216].

A number of dietary priorities have been identified in the literature and highlighted in this review that need to be addressed in order to improve dietary management of diabetes and NCDs in general in the MENA region. At the level of the individual, lack of knowledge and appreciation of the role of the dietitian in diabetic care are salient factors that negatively influence dietary behaviors and disease management and constitute a major barrier for effective self-management of diabetes in countries of the region [217].

Improving dietetic care for diabetes also requires greater investment in diabetes and nutrition research, and gathering region-specific and country-specific data on food consumption patterns, in addition to increasing healthcare expenditure which ranged between 3% and 7.6% in 2015 and was way lower than the world average of 9.9% [216].

Actions to improve dietetic care of diabetes in the region require political commitment and the creation of a clear research agenda that identifies knowledge gaps and informs policy makers about the most effective and most feasible action plans to mitigate the health, social, and economic burdens of diabetes and NCDs [212]. Recently, a few governments in the MENA region have devised several policies, programs and strategies to address the growing prevalence of diabetes. Consequently, new taxes, legislations, and government policies are driving demand for fiber-enriched, sugar-reduced, lower fat foods, and dairy alternatives, in order to combat lifestyle diseases. Examples of legislation across the region include the implementation of sin taxes on sugar and trans fats in a few countries such as Saudi Arabia, Kuwait, Oman, Qatar and UAE. Furthermore, it has been reported that consumers in the Middle East are becoming more aware of the relevance of healthy eating as a result of several educational programs that addressed the importance of lowering intake of trans fats and added sugars. One example of such programs is the Healthy Food Strategy introduced by the Saudi government in 2018 in order to promote healthy lifestyles in an effort to combat obesity and its complications, most notably T2DM [216].

Finally, further implementation of a comprehensive whole food system approach targeting the obesogenic environment and food supply chain is ought to be adopted in the MENA region to foster the adherence to the traditional Mediterranean diet as a cost-effective and sustainable dietary pattern against NCDs. The adoption of a whole food system approach requires concerted efforts from different stakeholders and sectors including individuals, policy makers, the food industry, and research communities to improve the quality of diabetic care in the region and mitigate its morbidity and mortality.

## 5. Conclusions

Dietary and lifestyle interventions and adhering to recommendations of specialized healthcare professionals are a cornerstone for the effective prevention and management of diabetes. Research to understand NCDs’ risk factors and optimize dietary advice to diabetics is ongoing and emerging evidence is challenging many longstanding notions. Over the past decade, dietary guidelines have moved away from focusing on single nutrients to studying the effects of whole foods, food groups or dietary patterns. Diets focusing on single nutrients or macronutrients whether they are carbohydrate, protein or fat are often associated with adverse effects and fail to consider the substituting nutrient, other nutrients’ interactions among each other and the source of foods, among other confounders. While nutrient-based research is crucial to uncover the mechanisms of actions underlying the effects of foods on health, a dietary pattern focused approach has been advocated to become the foundation for dietary guidelines as recommending intake of foods instead of nutrients is more readily translatable into practical advice [218]. The translation of nutrition guidelines into easy practical advice is especially important for individuals with diabetes who are required to have advanced skills of counting carbohydrate intake depending on their insulin dosage and reading nutrition labels [219]. Moreover, until today, the evidence to confirm the superiority of any particular macronutrient distribution over the other for the prevention and management of diabetes remains lacking. The Mediterranean and DASH diets are considered amongst the healthiest dietary patterns available and are almost consistently associated with health benefits. These diets largely consist of consuming fresh and unprocessed foods such as fruits, vegetables, nuts, legumes, whole grains and olive oil that are known to be associated with positive health outcomes, while limiting the consumption of unhealthy, often processed foods such as processed red meat and SSB. In the context of diabetes, the benefits of the Mediterranean and DASH diets specifically include prevention of diabetes, decreasing mortality and CVD risk and improving glycemic control, in addition to their positive association with weight loss in the obese [219,220].

In the MENA region, where the prevalence of obesity and NCDs are soaring, adherence to the native Mediterranean diet is eroding and challenged by rapidly occurring nutrition transition driven by economic growth, globalization, and urbanization. Relative to the available resources in rich countries of the region, efforts to halt the obesity and NCD epidemics are lagging and do not match the magnitude of the burdens. One of the key priority areas in the region to improve care for diabetes and other NCDs is the need to increase biomedical research investments in order to fill the gaps in region-specific data and nutrition research, as most of the current knowledge is extrapolated from the western part of the world. The potential role of dietetic care as cost-effective clinical and public health interventions is also far from being realized, challenged by the lack of awareness and appreciation of its evident effectiveness in the prevention and management of this disease at the levels of the public, healthcare providers, and governments. Scaling up the action against NCDs and obesity requires devising policies and strategies to better integrate dietetic services within the multi-disciplinary framework of care for diabetes, and increase their utilization by the public and referring physicians. While no single intervention can halt the rising NCD and obesity epidemics, sustained and consorted efforts from all stakeholders, greater political and fiscal commitments, and adopting a whole system approach that creates and fosters a healthy food environment and promotes the adherence to the region’s native Mediterranean diet are desperately needed.

## Figures and Tables

**Table 1:** Selected systematic reviews and meta-analyses comparing different dietary approaches in diabetes management.

Author	Sample Size	Duration	Outcomes	Comparison	Result
Ajala et al. (2013) [90]	3073 adults with T2D	≥6 months	Glycemic control; lipid profile; weight loss	7 dietary approaches (LC, V, vegan, low GI, HF, MD. HP) vs. control diets	MD, LC, HP and low GI all improved glycemic control MD showed largest effect size MD and LC showed greater weight loss All diets increased HDL except HP
Jannasch et al. (2017) [91]	16 study populations (non-diabetic participants)	-	Diabetes incidence	MD, DASH, HEI, AHEI	MD, DASH and AHEI showed great potential for diabetes prevention
Schwingshackl et al. (2018) [92]	4937 adults with T2D	≥12 weeks	HbA_1_c (%); fasting blood glucose (mmol/l)	9 dietary approaches (LF, LC, MC, HP, MD, low GI/GL and PD vs. control)	LC achieved greatest HbA_1_c reduction (SUCRA: 84%) MD achieved best results for fasting blood glucose (SUCRA: 88%)
Neuenschwander et al. (2019) [93]	5360 adults with T2D	≥12 weeks	LDL-C (mmol/l); HDL-C (mmol/l); TG (mmol/l)	9 dietary approaches (LF, LC, MC, HP, MD, low GI/GL and PD vs. control)	MD was the most effective to manage diabetic dyslipidemia (SUCRA: 79%).
De Carvalho et al. (2019) [94]	Adults with T2D	8 weeks–4 years	HbA_1_c (%)	Dietary patterns favoring glycemic control vs. control diets	Vegan, V, MD and DASH achieved greatest reduction
Abbasnezhad et al. (2020) [95]	1130 adults with T2D	2 weeks–3 years	Systolic and Diastolic BP	11 dietary approaches (Vegan, LF, LS, HF, LP, HP, LC, Low GI, PD, MD, Korean traditional diet)	LS achieved greatest reduction for systolic BPHF achieved greatest reduction for diastolic BP

T2D: Type 2 diabetes; LDL-C: low-density lipoprotein cholesterol; HDL-C: high-density lipoprotein cholesterol; TG: triglycerides; BP: blood pressure; WC: waist circumference; LF: low fat; MD: Mediterranean diet; HP: high protein; LP: low protein; LC: low carbohydrate; MC: moderate carbohydrate; V: vegetarian; PD: paleolithic diet; GI: glycemic index; GL: glycemic load; LS: low sodium; HF: high fiber; DASH: Dietary Approach to Prevent Hypertension; HEI: Healthy Eating Index; AHEI: Alternative Healthy Eating Index; SUCRA: surface under cumulative ranking curve.

**Table 2:** Selected systematic reviews and meta-analyses on association of dietary fat with diabetes outcomes.

Author	Sample Size	Follow-up	Objective	Result
De Souza et al. (2015) [173]	-	-	Association of fat intake with mortality, CVD and T2D	SFA not associated with all-cause mortality, CVD mortality, total CHD mortality, ischemic stroke or T2D Total TFA associated with all-cause mortality, CHD and CHD mortality but not ischemic stroke or T2D Industrial but not ruminant TFA associated with CHD and CHD mortality Ruminant TFA inversely associated with T2D
Imamura et al. (2016) [176]	4220 adults with T2D	3–166 days	Effects of fat intake on blood glucose, insulin, HbA1c, insulin sensitivity, and insulin secretion	Isocaloric substitution of SFA and carbohydrates with PUFA significantly improved fasting glucose and HOMA-IR, but not fasting insulin
Schwingshackl et al. (2017) [177]	187,068 adults with or without T2D	2 weeks–22 years	Association between intake of olive oil and glycemic control	Highest versus lowest intake of olive oil associated with 16% decreased T2D risk
Wanders et al. (2019) [178]	576 adults with and without T2D	3–16 weeks	Effects of plant-derived PUFA on fasting glucose, fasting insulin, HOMA-IR, HbA1c, post-challenge measures of glucose metabolism and markers of insulin sensitivity	-Isocaloric substitution of SFA or carbohydrates with PUFA reduced fasting insulin and HOMA-IR, but not glucose -Highest PUFA intake associated with larger effects on fasting insulin and HOMA-IR
Brown et al. (2019) [179]	-	≥24 weeks	Effects of dietary fat intake on diabetes diagnosis, fasting glucose, fasting insulin, HbA1c, HOMA-IR	T2D incidence associated with omega 6 FA and inversely associated with higher linoleic acid and
Jiao et al. (2019) [180]	11,264 adults with T2D based on 2 cohort studies	Cohort 1: 1980–2014 Cohort 2: 1984–2014	Association between dietary fat intake and mortality	-PUFA associated with lower CVD and total mortality -PUFA n-3, linoleic acid associated with lower total mortality -MUFA of animal but not plant origin associated with greater total mortality -Replacing 2% of energy from SFA with PUFA or linoleic acid associated with 13% and 15% decreased CVD mortality respectively and 12% decreased total mortality for PUFA
Neuenschwander et al. (2020) [181]	53,185 adults	4.1 years–32 years	Association between intake of different types of dietary fat and T2D incidence	-Vegetable fat (PUFA, plant-based linoleic acid) associated with lower T2D incidence at low doses -Animal derived long chain omega 3 FA associated with increased T2D incidence -SFA, total omega 3 FA, trans FA and MUFA not associated with T2D incidence

T2D: type 2 diabetes; FA: fatty acids; SFA: saturated fatty acids; MUFA: monounsaturated fatty acids; PUFA: polyunsaturated fatty acids; TFA: trans fatty acids; HOMA-IR: homeostatic model assessment for insulin resistance; CHD: coronary heart disease; CVD: cardiovascular diseases.
